# The nurse practitioner ethicist: Distinct from a nurse ethicist?

**DOI:** 10.1177/09697330251350386

**Published:** 2025-06-10

**Authors:** Jesse Michael Kay

**Affiliations:** 506057 Baylor College of Medicine

**Keywords:** nurse practitioner, nurse ethicist, nursing ethics, virtue ethics, clinical ethics, nurse practitioner ethicist

## Abstract

Ethics has been central to the nursing profession. Challenges in patient care have arisen through advances in medicine through science and technology. These advances have led to patients being sustained in ethically difficult situations. Nurses have uniquely witnessed both the suffering of patients and rendered care for extended periods of time at the bedside. Through these caring relationships nurses have developed expertise in advocating for their patients. Many nurses have also returned to graduate school to develop their nursing science and ethical reasoning. Recently, the field of nursing has developed the role of the nurse ethicist. Nurse ethicists often also work as clinical ethics consultants. Additionally, nursing has advanced to include nurse practitioners as advanced practice nurses. Nurse practitioners have also obtained additional education in ethical reasoning and are working in roles similar to nurse ethicists and clinical ethicists. Given the science and nature of the nurse practitioner there may be unique facets to nurse practitioners who are ethicists. To date there are no proposals for nurse practitioner ethicists. Let this be the first proposal addressing the distinctions of the nurse practitioner ethicist.

## Introduction

Human beings fall ill and require the expertise of clinicians, including physicians, nurses, nurse practitioners, and all healthcare professionals who immediately care for patients at the bedside. Typically, nurses care for these patients through a nurturing relationship focused on healing and health promotion. In agreement, Jonsen, Siegler, and Winslade believe that the “central feature of the clinical encounter is the therapeutic relationship between clinicians and patients—a relationship that is imbued with ethical responsibilities.”^1^ Through these relationships, ethical concerns may arise due to the advancement of science, medicine, and technology. This necessitates that clinicians either have additional ethics training beyond their entry level studies for their profession or that they have access to a clinical ethics consultant.

Recently, nurses have obtained graduate education in bioethics, ethics, or philosophy and are developing the new role of the nurse ethicist. But there is currently no literature on the similarities or differences of an advanced practice nurse practitioner in this role. Should the nurse practitioner practice as a nurse ethicist or a nurse practitioner ethicist? Is there something distinct about nurse practitioners practicing as ethicists? This paper will focus on proposing a particular view of a nurse practitioner ethicist by defining the role of the ethics consultant, the role of the nurse ethicist, how the internal morality of nursing and medicine and virtue theory guide the nurse practitioner, and what makes a nurse practitioner ethicist distinct from ethics consultants and nurse ethicists.

## The ethics consultant

To discover if there are differences or similarities between a nurse practitioner ethicist and an ethics consultant, it is important to define what an ethics consultant is and what an ethics consultant does. Ethics consultation is “a set of services provided by an individual or group in response to questions from patients, families, surrogates, health care providers, or other involved parties who seek to resolve uncertainty or conflict regarding value-laden concerns that emerge in health care.”^2^ In other words, an ethics consultant is a professional who aims to “improve the quality of health care through the identification, analysis, and resolution of ethical questions.”^3^ Of note, the professionalization of the ethics consultant role continues to have variability internationally. For the purpose of this discussion, the American Society of Bioethics and Humanities and American literature will be the focus. With advances in medicine and technology ethical questions arise more often, and clinicians are faced with decisions without a graduate education or background in advanced ethical reasoning. This is why ethics consultants are requested to assist in ethically challenging cases during patient care.

The primary goal of the ethics consultant is not to decide the course of action for the patient or the healthcare team but rather to facilitate resolution.^4^ To accomplish this end, the consultant meets and discusses goals with the healthcare team, and discusses the patient’s goals and values with the healthcare team, patient, or surrogate.^5^ It is worthy to note that sometimes patients or surrogates are not involved in conversations that the ethicist resolves with the healthcare team, but the patient or surrogate are often involved. While gathering information from the healthcare team, the consultant contextualizes the patient’s medical situation, needs, and limitations. Afterward, the consultant may meet with the patient or the surrogate to determine what the patient’s social, cultural, and religious beliefs are as well as their personal preferences regarding the situation. During this process, the consultant reviews relevant laws and hospital policies that may affect the case. After obtaining all of this information, the ethics consultant reviews the ethical question and applies ethical theories, moral reasoning, and ethical principles to assist with decision-making. Throughout the consultation, the ethics consultant helps “clarify the facts of the case,” “help[s] stakeholders express their views and concerns,” “improve[s] communication,” “provide[s] emotional support,” and “negotiate[s] an acceptable resolution.”^4(pp127–128)^ In short, the ethics consultant attempts to bring the healthcare team and the patient or surrogate together to resolve the ethical dispute, but provides ethical recommendations regardless of if the parties come to consensus. After reviewing the role of the ethics consultant, it is now time to see how the role of the nurse ethicist differs.

## A particular view of the nurse ethicist and the internal morality of nursing

What is a nurse ethicist and how does the nurse ethicist differ from an ethics consultant? To answer this question, a brief acknowledgment of a particular view of nursing ethics must first be discussed prior to describing what a nurse ethicist is and what makes him or her distinct from an ethics consultant. While the history of nursing ethics is important, it is beyond the scope of this article to do justice to its history. With that said, nursing ethics is about the primacy of the nurse–patient relationship. Even though medical ethics has the same primary focus, for now the discussion will center on the nurse–patient relationship. It is this focus on relationship with the patient where nursing ethics obtains its morals internal to the practice of nursing. The internal morality of nursing identifies that nurses care for patients from the theme of service. According to Fowler, from service arises the principles of devotion to the patient, loyalty to other clinicians, and assistance to society.^6^ By service, what is meant is that nurses act in service of the good of their patients. This does not mean that nurses act as servants of patients but rather that they act to promote the good of their patients originating from the notion of service, which many religions emphasize as giving back to their fellow humans without expectations of personal gain in return. An example would be, “It is more blessed to give than to receive.” (Acts 20:35 ESV).

Out of the nurse–patient relationship and theme of service, the nurse practices the principle of devotion to the patient.^6,7^ Through devotion to the patient, the nurse exercises confidentiality and fidelity to the patient to first advocate for the patient’s good and second for the good of society as a whole.^6,7^ While devotion is limited to patients, the nurse is loyal to other clinicians to unify their team efforts to promote the patient’s good. Even though nurses are loyal to other clinicians, and devoted to patients, nurses still exercise their moral conscience to excuse themselves from unethical practices, and they also push for social change to benefit future patients.^6,7^ It is important to note that while the nurse practices devotion to the patient, the nurse is protected by the principle of service to the patient’s good to prevent misuse of the nurse’s role. At times patients may ask nurses to compromise their commitment to the patient’s good in exchange for the patient’s personal preferences that may conflict with nursing’s commitment to promotion of the patient’s good. While it is important to honor the patient’s personal preferences, nursing does not promote consumerism but rather a relationship that is purposed to benefit the patient without compromising nursing’s commitment to the patient’s good of being a human being. If patients violate their own personal values and they also compromise the practice of nursing, then it is permissible for the nurse to politely deny the request, discuss it further with the patient, and to transfer the care of the patient to another nurse if needed. It is from service to the patient’s good, devotion to the patient, loyalty to other clinicians, and assistance to society that the nurse ethicist considers how this commitment internal to nursing’s moral practice shapes how they ought to respond.

Now that the internal morality of nursing has been discussed, what makes a nurse a nurse ethicist and different from a clinical ethicist? Various authors have differing opinions on what makes a nurse a nurse ethicist. It will be helpful to express what some generally agree upon and connect what likely this view of nursing ethics would support as a role for a nurse ethicist. Some authors believe that educational and experience requirements are important for nurse ethicists.^8,9^ Additionally, a few agree that a nurse ethicist should have clinical experience as a nurse, masters or doctoral education in both nursing and ethics, and have knowledge in clinical ethics obtained either by certification and practice or by a clinical fellowship.^8,9^ Typically, nurses do not obtain a significant amount of education in moral reasoning, moral philosophy, or applied ethics. To acquire relevant education, most nurse ethicists obtain a masters or doctorate degree in nursing and a masters or doctorate degree in ethics, bioethics, or philosophy. Once these relevant qualifications are acquired, what makes a nurse ethicist distinct and what is their role?

The major distinction from other medical ethical fields is that the unique nature of the nurse–patient relationship is the most important factor that sets nursing ethics apart.^9–11^ While all healthcare professions value their relationship with the patient, the nurse–patient relationship is unique for a few reasons. First, Pilkington and Giuliante note that “throughout nursing education and generally in every career path that follows, creating nurturing relationships is emphasized. Compassion and respect for the dignity of every patient is the framework upon which these relationships are built.”^11(p672)^ One could argue that many professions also support this notion, but, as noted above, nursing ethics uniquely emphasizes service and devotion to the patient’s good and it can be argued that the nurse–patient relationship is central to nursing’s practice.^11^

Another notable reason that the nurse–patient relationship is key for the nurse ethicist is that nursing educates and focuses on creating deep relationships with patients and families relatively quickly through trust.^11^ This focus on relationship building through trust allows the nurse to learn the desires, goals, and values of the patient and their family to advocate on their behalf. Pilkington and Giuliante believe that this trust not only allows the nurse to establish “meaningful relationships,” but also allows “the opportunity to take notice of burgeoning ethical quandaries that may be otherwise overlooked in mainstream ethics discourse.”^11(p676)^ Essentially, the nurse ethicist has a deep commitment to patients and the internal morality of nursing which guides them to place deep value in relationships and assists in mitigating ethical issues before they become worse potentially through policy revision or creation. The nurse ethicist is distinct from the commitment of the profession to relationships, from the expertise found in building rapid relationships through experience practicing as a clinical nurse, and from the expertise of clinical experience in navigating health systems to serve patients and prevent moral dilemmas.^10,11^

So what is a nurse ethicist and what makes them different than an ethics consultant? Grace and Milliken assert that the “title nurse ethicist denotes a person who has both nursing and ethics expertise and sees these as in some ways inseparable from the work in which they are engaged.”^12(p663)^ In other words, a nurse ethicist practices ethics from the internal morality of nursing and is usually also an educator, author, clinical ethics committee member, and clinical ethics consultant. Morley, Robinson, and Wocial point out that “nurse ethicists approach ethics consultation from the hermeneutic of nursing—a relationship centered approach specific to the practice of nursing.”^9(p691)^ This means that a nurse ethicist has the advantage of extensive clinical experience practicing nursing, has expertise in nursing ethics and nursing science, has experience navigating healthcare systems, has experience in building rapid relationships with families and patients, and knows the ethical duties and virtues required of nurses. A non-nurse clinical ethicist, Ford, believes that nurse ethicists have an advantage over other clinical ethicists because they “tap into nursing culture,” have medical credibility as ethicists from their clinical experience, and because they “identify needs and solutions with an eye from the inside while still maintaining a professional role on the outside as an ethicist.”^13(pp654–655)^ In short, a nurse ethicist is a nurse who is an ethicist of clinical and medical ethics as well as nursing ethics who practices ethics from a commitment to the internal morality of nursing which places primacy in the nurse–patient relationship.

## The internal morality of the nurse practitioner

Now that an ethics consultant and a particular view of a nurse ethicist have been defined, it is now important to define what a nurse practitioner is to see how a nurse practitioner ethicist is informed by the morality internal to their practice. Nurse practitioners became a profession in 1965 when a pediatrician, Henry Silver, and a pediatric public health nurse, Loretta Ford, identified that underserved children needed further care in their community.^14^ Their intention was to create the nurse practitioner (NP) role to educate nurses in medical diagnostic reasoning and primary care from the guidance of the nursing model:

Nurse practitioners require education for sophisticated nursing knowledge, deep expansive skillsets, knowledge of medicine, and related scientific disciplines to support higher-level clinical and diagnostic reasoning within a broader scope of nursing practice.^15(p52)^

As noted by Peterson and Potter, “NPs are and always will be nurses, but they possess unique skills and have a unique role that sets their profession apart.”^16(p120)^ Because of their unique skills, nurse practitioners are educated from both nursing and medical models so that they can safely and competently examine, diagnose, and treat patients.^15^ This means that nurse practitioners utilize their nursing education and new medical and nursing knowledge to practice advanced practice nursing. This does not mean that they practice medicine. Nurse practitioners practice from a commitment to nursing, as discussed above, and are also held accountable to the medical ethics that physicians are required to practice due to the changes and advancement of nursing practice. It is critical to acknowledge the caution that Wood makes when she alerts NPs to practice from advanced practice nursing and not solely from medicine: “When NPs abandon nursing knowledge by practicing *solely from the medical model*, rather than the nursing model, they risk becoming physician extenders.”^15(p55)^

Today, the nurse practitioner role has expanded to include inpatient and outpatient settings across the lifespan. Nurse practitioners evaluate, diagnose, prescribe, treat, and promote health with additional masters or doctoral education in medical knowledge and diagnostic reasoning through the lens of the nursing model. But what makes a nurse practitioner virtuous or ethical in their practice and how does this inform a nurse practitioner ethicist’s role? To answer this question, it is important to discuss that a nurse practitioner practices the internal morality of nursing and also must have a commitment to honor the internal morality of medicine which holds accountable those who diagnose, prescribe, treat, and promote health. This combination of the internal morality of nursing with honoring the internal morality of medicine leads to a particular view of the internal morality of the advanced practice nurse practitioner that will be put forward. Both the commitment and honor focus on the centrality of the nurse practitioner–patient relationship that the author believes could be well informed by virtue ethics.

As the proposed particular view of nursing ethics reveals that the internal morality of nursing is that of the nurse–patient relationship through service, it is now important to define the internal morality of medicine and how the nurse practitioner is held accountable and informed by both. Similarly to nursing ethics, physician and clinical ethicist Edmund Pellegrino emphasized that the clinician–patient relationship is the center of the internal morality of medicine.^17^ Through this relationship medicine teaches that it is purposed to “heal the sick, to protect and nurture health, [and] to maintain and restore physical well-being.”^18(p144)^ In essence, the healthcare relationship focuses on healing and helping patients and places this relationship above the clinician’s self-interests.^19^

In order to avoid promoting the clinician’s self-interest through paternalism and to avoid reducing healthcare to consumerism and clinicians to technicians, Pellegrino asserted that healthcare professionals should practice beneficence-in-trust. This view internal to medicine’s practice progresses the patient’s good by honoring the patient’s preferences while limiting the patient’s choices to only those permitted in healing and helping relationships.^17^ These limits are found in the Hippocratic oath which states that the aim of medicine is “to help the sick…never with the intention of doing harm.”^18,20(p67)^ Moreover, Hippocrates stated that medicine is purposed for the “removal of the distress of the sick, the alleviation of the more violent diseases and the refusal to undertake to cure cases in which the disease has already won the mastery, knowing that everything is not possible to medicine.”^21(p140)^ Therefore the internal morality of medicine is the clinician–patient relationship that recognizes medicine’s *telos* as healing and helping, and when healing is no longer able, then it focuses on helping through caring. Thus, the nurse practitioner practices the internal morality of nursing and honors the internal morality of medicine by practicing a healing and helping relationship stewarded by this *telos* of healthcare with a primary focus on service to the patient’s good.

## The internal morality of advanced nursing practice and virtue ethics

Now that the focus of relationships is seen to be internal to the morality of the nurse practitioner’s practice, how does the nurse practitioner carry out these good aims virtuously and how does this translate to the role of a nurse practitioner ethicist? Informed from the internal morality of nursing while honoring medicine’s internal morality, this philosophy of advanced nursing practice could encourage nurse practitioners to utilize these aspects intrinsic to their profession with virtue ethics to practice ethically. As Thomasma and Pellegrino once said, “virtue and principle-based theories in medical ethics must be closely linked with the nature of medicine itself that is, with a philosophy of medicine.”^22(pxii)^ Given that this particular view encourages nurse practitioners to practice the philosophy intrinsic to both nursing and medicine, their nature of advanced nursing practice also uses the principles of beneficence, non-maleficence, autonomy, and justice informed by the internal morality of the profession through the *telos* of healing and helping via service to the patient’s good.

But how does principlism properly combine with a virtue theory informed by the philosophy of advanced nursing practice even though both principlism and virtue ethics have weaknesses? Many have noted that principles do not help guide the actions of clinicians as they “lack any systematic relationship to each other, and they conflict with each other.”^23(p219)^ Clouser and Gert noted that a “moral theory…is needed to unify all the ‘considerations’ raised by the ‘principles’ and thus to help us determine what is appropriate.”^23(p228)^ For the nurse practitioner ethicist, this theory to unify these principles could be virtue ethics informed by the internal morality of advanced nursing practice, or a teleologically based philosophy of advanced nursing practice, as described above. But if virtue ethics and principlism were alone without the internal morality of the profession, then it would fall prey to the weakness of virtue ethics. This weakness is that only a virtuous person can practice virtuously. But how would one practice virtuously if they are not yet virtuous? And how would they know what a virtuous nurse practitioner is? This is why the internal morality of advanced nursing practice is needed to define what is intrinsic to the virtuous nurse practitioner’s practice. Thus, the argument of this paper’s author is that the virtuous nurse practitioner practices the art of nursing with an honor to the internal morality of medicine found in the philosophy of advanced nursing practice.

Now that the internal morality of advanced nursing practice has been defined and the importance of virtue theory to assist with ethical decision-making in advanced practice, what are the virtues central to the nurse practitioner–patient relationship? Some of these virtues include trust, compassion, temperance, fortitude, justice, integrity, and prudence. As shown above, both the internal morality of nursing and medicine find trusting relationships foundational to good patient care. The nurse practitioner establishes trust rapidly with their patients from their history of nursing and practices beneficence-in-trust to promote the patient’s autonomy tempered by the good aims of the philosophy of advanced nursing practice. A virtuous nurse practitioner should demonstrate compassion for their patients as they are vulnerable while seeking care. The nurse practitioner committed to the internal morality of advanced practice would have the fortitude, or moral courage, to advocate for their patients in service to the patient’s good to ensure that patients can receive the interventions or palliative care that they need regardless of the cost to the nurse practitioner’s station within their institution. This fortitude is needed when nurse practitioners advocate for justice or equal treatment of their patients in comparison to others.

The final two virtues, integrity and prudence, perhaps hold the most weight for the nurse practitioner in maintaining devotion to the patient’s good in a healing and helping relationship. Integrity refers to both the virtue of the nurse practitioner’s character to act rightly in all situations as well as the nurse practitioner respecting the natural integrity of the mind and body of patients as persons.^22^ In alignment with integrity of patients as persons, Curlin and Tollefsen purport that health and human flourishing in healthcare is promoting the natural homeostasis of human beings which is “‘the well-working of the organism as a whole’, realized and manifested in the characteristic activities of the living body in accordance with its species-specific life-form.”^24(p31)^ In other words, the virtuous nurse practitioner is limited to only rendering healing and helping care to patients if they honor the natural integrity of the “bodily, psychosocial, and intellectual elements of their lives.”^22(p129)^ This integrity implements all of the virtues to honor the healing and helping nurse practitioner–patient relationship to maintain trust that the nurse practitioner will not practice against the internal morality of advanced nursing practice to harm the vulnerable.

Finally, prudence is the ordering virtue for the nurse practitioner as it brings together wisdom, or practical reason, with principles, duties, and virtues. Prudence “enables us to arrive at the right and good ordering of principles and concrete facts in particular cases.”^22(p23)^ The nurse practitioner who is prudent “can order habitually fact and principle most sensitively and correctly to each other and act appropriately to achieve the good for the patient.”^22(p23)^ Prudence is necessary in order to apply the virtues and philosophy of advanced nursing practice to sort through beneficence, non-maleficence, autonomy, and justice to make the right and the good decision to help patients. Prudence guides the nurse practitioner to choose the morally good action in the interests of the patient’s good through beneficence-in-trust. But what is the patient’s good?

Pellegrino and Thomasma state that there are four philosophical goods for patients. There is the patient’s medical good, the patient’s opinion of his or her own good, the good for the patient based on what is good for “humans as humans and members of the human community,” and there is the spiritual good of the patient.^22(p58)^ When making decisions, the prudent nurse practitioner sorts through the virtues, principles, and internal morality of advanced nursing practice to make appropriate recommendations for treatments that would honor the patient’s wishes without violating the limitations practically placed by the healing and helping relationship focused on devotion to the patient’s good. This means that the nurse practitioner and patient should discuss these four goods to make the most appropriate decision as each decision may greatly affect the patient’s life. If the nurse practitioner or patient does not agree, then they may end their relationship after transferring the patient’s care to another clinician. But it is imperative that nurse practitioners and patients recognize that the medical good is not always the highest good, and that the patient’s opinion of the good may not be the highest good either. Ultimately, the temperance of these decisions, as shown by the integrity of the patient as person above, reveals that the two highest goods are the patient’s good as a human being and the patient’s spiritual good. Prudent nurse practitioners and patients utilize the four goods to conclude what may be the morally good action in the patient’s interest. It is apparent that this view is limited only to those nurse practitioners who wish to practice from this particular view of the internal morality of advanced nursing practice informed by virtue ethics. But in short, this view proposes that the virtuous nurse practitioner, and nurse practitioner ethicist, centers their practice through prudence to engaging in healing and helping relationships with patients out of service to the patient’s good.

## The nurse practitioner ethicist: Distinct

After defining the roles of the clinical ethics consultant and nurse ethicist as well as the internal morality of advanced nursing practice that shape the virtuous nurse practitioner through virtue ethics, it is finally time to elucidate the distinctions of the nurse practitioner ethicist according to this proposal. To begin, this proposal asserts that a nurse must first practice as a clinical nurse and then obtain advanced graduate education in nursing science with a focus on nurse practitioner studies. In order to become a nurse practitioner ethicist, the nurse practitioner must practice clinically and also either obtain a masters or doctorate degree in bioethics, ethics, or philosophy, or graduate from a clinical ethics fellowship to be adept at moral reasoning and clinical ethics beyond the typical nursing graduate education. Just as the nurse ethicist practices from commitment to the internal morality of nursing, the nurse practitioner ethicist practices from the internal morality of nursing while being held accountable to the internal morality of medicine because of the nature of their advanced practice requiring medical knowledge to diagnose, prescribe, and treat. The author of this article terms this particular view as the internal morality of advanced nursing practice or the philosophy of advanced nursing practice. This commitment to the internal morality of advanced nursing practice gives nurse practitioner ethicists a unique perspective to only provide ethical recommendations that align with the healing and helping relationships found in nursing and medicine.

Additionally, this view purports that the nurse practitioner ethicist should have practiced both as a clinical nurse and as a clinical nurse practitioner which gives expertise in both rendering bedside care and in medical decision-making. This provides the nurse practitioner ethicist with the same distinctions as nurse ethicists including nursing clinical experience, expertise in nursing ethics and science, experience in navigating healthcare systems, experience in building rapid relationships with families and patients, and knowing the ethical duties and virtues required of nurses and now also nurse practitioners. The distinct role of nurse practitioner ethicist also provides the advantage of these same nurse ethicist distinctions from the lens, training, and experience of a nurse practitioner that a nurse cannot relate to due to the unique role of nurse practitioners.^16^ The nurse practitioner ethicist is also distinct because they can relate to and assist other nurse practitioners who experience moral distress or moral challenges that are unique to the nurse practitioner profession. The advanced practice role of a nurse practitioner ethicist also provides additional expertise in nurse practitioner science, pathophysiology, pharmacology, medical decision-making, and team-based care via collaboration with physicians and other nurse practitioners. This role adds to the nurse ethicist with additional education and experience to prioritize the good of the patient and contextualize the four goods for the patient in ways that other healthcare team members may not be able to. This is due to the unique life experience and role of nurse practitioners having been both bedside nurses and advanced practice nurses. This provides the nurse practitioner ethicist with the weighty perspective of medical decision-making tempered by their commitment to nursing and service to the patient’s good.

As shown earlier, the author of this article believes that the nurse practitioner ethicist might best practice from the philosophy of advanced nursing practice through virtue ethics. This would frame ethical recommendations from the teleology of nurse practitioner practice through virtue ethics focusing on what the virtuous nurse practitioner would do. This philosophy of moral reasoning could work well because the internal morality of medicine is to help and to heal through clinician–patient relationships and the internal morality of nursing is to serve the patient’s good in the nurse–patient relationship. This could be best accomplished through the earlier proposed virtues guided by nurse practitioner integrity, the patient’s integrity as a person, and by prudence. Fortunately, this philosophy of advanced practice nursing focuses on the nurse practitioner–patient relationship and provides limitations to prevent the nurse practitioner from becoming simply a provider of services or from harming the patient. In summary, the nurse practitioner ethicist is distinct because he or she practices ethics from the internal morality of advanced nursing practice with additional training and experience in advanced nursing practice and ethics to provide a unique perspective while also being an educator, author, clinical ethics committee member, clinical ethics consultant, and nurse ethicist.

## Conclusion

This paper is the first to propose a unique view of the nurse practitioner ethicist and its distinctions. It focused on defining the ethics consultant, the nurse ethicist, how the internal morality of nursing and medicine and virtue theory could guide the nurse practitioner, and what makes a nurse practitioner ethicist distinct. It is important to note that while nurse ethicists and nurse practitioner ethicists have unique strengths that they add to teams, it is also important to have a diversity of perspectives on clinical ethics committees and ethics consultation services. No one role is superior to another but can add something unique to assist patients. Nurse practitioner ethicists can add rare viewpoints to teams and perhaps unify physician and nursing teams to serve the good of patients further. Afterall, most clinical ethics teams in the United States of America are largely formed by physicians and nurses.^25^ Future work should focus on further development of the nurse ethicist, the nurse practitioner ethicist, the internal morality of advanced nursing practice or the philosophy of advanced nursing practice, the intersection of virtue ethics with the philosophy of advanced nursing practice, and the spiritual and religious views that may lend strength to the philosophy of advanced nursing practice.

## Data Availability

The author encourages citations and further development of thoughts presented in this article.*

## References

[1] Jonsen A Siegler M Winslade W . Clinical ethics: a practical approach to ethical decisions in clinical medicine. 9th ed. New York, NY: McGraw Hill, 2022.

[2] American Society for Bioethics and Humanities’ Core Competencies Update Task Force . Core competencies for health care ethics consultation: the report of the American society for bioethics and Humanities. 2nd ed. Chicago, IL: American Society for Bioethics and Humanities, 2011. 10.1080/15265161.2012.750388 23391049

[3] Tarzian A and the ASBH Core Competencies Update Task Force . Health care ethics consultation: an update on core competencies and emerging standards from the American society for bioethics and Humanities’ core competencies update task force. AJOB 2013; 13: 4. 23391049 10.1080/15265161.2012.750388

[4] Lo B . Resolving ethical dilemmas: a guide for clinicians. 6th ed. Alphen aan den Rijn: Wolters Kluwer, 2020, pp. 126–129.

[5] Campelia G Dudzinski D . Ethics consultation mission, vision, goals, and process. In: Hester Schonfeld (eds) Guidance for healthcare ethics committees. 2nd ed. Cambridge: Cambridge University Press, 2022. pp. 62–64.

[6] Fowler M . Why the history of nursing ethics matters. Nurs Ethics 2017; 24: 292–304. 28511609 10.1177/0969733016684581

[7] Fowler M Schoonover-Shoffner K . Rising to ‘the highest morals’: the rich history of nursing ethics. J Christ Nurs 2023; 40: 86–95. 36872538 10.1097/CNJ.0000000000001039

[8] Johnstone M . Nurse ethicist: innovative resource or ideological aspiration? Nurs Ethics 2023; 30: 680–687. 37946394 10.1177/09697330231191817

[9] Morley G Robinson E Wocial L . Operationalizing the role of the nurse ethicist: more than a job. Nurs Ethics 2023; 30: 688–700. 37946392 10.1177/09697330221147898

[10] Wolfe I . Beyond the consult question: nurse ethicists as architects of moral spaces. Nurs Ethics 2023; 30: 710–719. 37946395 10.1177/09697330231151351

[11] Pilkington B Giuliante M . Nursing ethics as a distinct entity within bioethics: implications for clinical ethics practice. Nurs Ethics 2023; 30: 671–679. 37946388 10.1177/09697330231174535

[12] Grace P Milliken A . A semantic exploration: nurse ethicist, medical ethicist, or clinical ethicist: do distinctions matter? Nurs Ethics 2023; 30: 659–670. 37946385 10.1177/09697330221146251

[13] Jones J Ford P Birchley G , et al. Perspectives on the role of the nurse ethicist. Nurs Ethics 2023; 30: 652–658. 37946393 10.1177/09697330231189034

[14] Brennan C . Tracing the history of the nurse practitioner profession in 2020, the year of the nurse. J Pediatr Health Care 2020; 34: 83–84. 32063262 10.1016/j.pedhc.2019.12.005

[15] Wood S . Keeping the nurse in the nurse practitioner: returning to our disciplinary roots of knowing in nursing. Adv Nurs Sci 2020; 43: 50–61. 10.1097/ANS.0000000000000301 31922983

[16] Peterson M Potter R . A proposal for a code of ethics for nurse practitioners. J Am Acad Nurse Pract 2004; 16: 116–124. 15130066 10.1111/j.1745-7599.2004.tb00382.x

[17] Pellegrino E . Medical ethics: entering the Post-Hippocratic era. J Am Board Fam Pract 1988; 1: 230–237. 3223343

[18] Verhey A . The doctor’s oath – and a Christian swearing it. Linacre Q 1984; 51: 139–157. 11650681

[19] Pellegrino E . The philosophy of medicine reborn: a Pellegrino reader. 1st ed. Notre Dame, IN: University of Notre Dame, 2011.

[20] The oath. Lloyd GER (ed) Hippocratic writings. 1st ed. London: Penguin Classics, 1983.

[21] Hippocrates . The science of medicine. In: Llyod GER (ed) Hippocratic writings. 1st ed.. London: Penguin Classics, 1983.

[22] Pellegrino E Thomasma D . The virtues in medical practice. 1st ed. New York: Oxford University Press, 1993.

[23] Clouser K Gert B . A critique of principlism. J Med Philos 1990; 15: 219–236. 2351895 10.1093/jmp/15.2.219

[24] Curlin F Tollefsen C . The way of medicine: ethics and the healing profession. Notre Dame, IN: University of Notre Dame Press, 2021.

[25] Fox E Myers S Pearlman R . Ethics consultation in United States hospitals: a national survey. AJOB 2007; 7: 13–25. 10.1080/15265160601109085 17366184

